# Supplementary material for: Polarization and Dielectric Properties of BiFeO3-BaTiO3 Superlattice-Structured Ferroelectric Films
Source: Nanomaterials (Basel). 2021 Jul 19;11(7):1857. doi: 10.3390/nano11071857 (PMC8308477; doi:10.3390/nano11071857)
Supplement: Supplementary file 1 [file nanomaterials-11-01857-s001.zip › nanomaterials-1287380-supplementary.pdf]

nanomaterials

Article

Supplementary Materials of "Polarization and Dielectric Properties of $BiFeO_3$-$BaTiO_3$ Superlattice-Structured Ferroelectric Films"

Yuji Noguchi [1,*] and Hiroki Matsuo [2,*]

1 Division of Information and Energy, Faculty of Advanced Science and Technology, Kumamoto University, 2-39-1, Kurokami, Chuo-ku, Kumamoto 860-8555, Japan
2 International Research Organization for Advanced Science & Technology (IROAST), Kumamoto University, 2-39-1, Kurokami, Chuo-ku, Kumamoto 860-8555, Japan
* Correspondence: yuji19700126@gmail.com (Y.N.); matsuo_h@cs.kumamoto-u.ac.jp (H.M.)

Citation: Noguchi, Y.; Matsuo, H. Polarization and Dielectric Properties of $BiFeO_3$–$BaTiO_3$ Superlattice-Structured Ferroelectric Films. *Nanomaterials* **2021**, *11*, 1857. https://doi.org/10.3390/nano11071857

Academic Editor: Dong-Joo Kim

Received: 21 June 2021
Accepted: 16 July 2021
Published: 19 July 2021

Publisher's Note: MDPI stays neutral with regard to jurisdictional claims in published maps and institutional affiliations.

Copyright: © 2021 by the authors. Licensee MDPI, Basel, Switzerland. This article is an open access article distributed under the terms and conditions of the Creative Commons Attribution (CC BY) license (https://creativecommons.org/licenses/by/4.0/).

Supplementary Figures

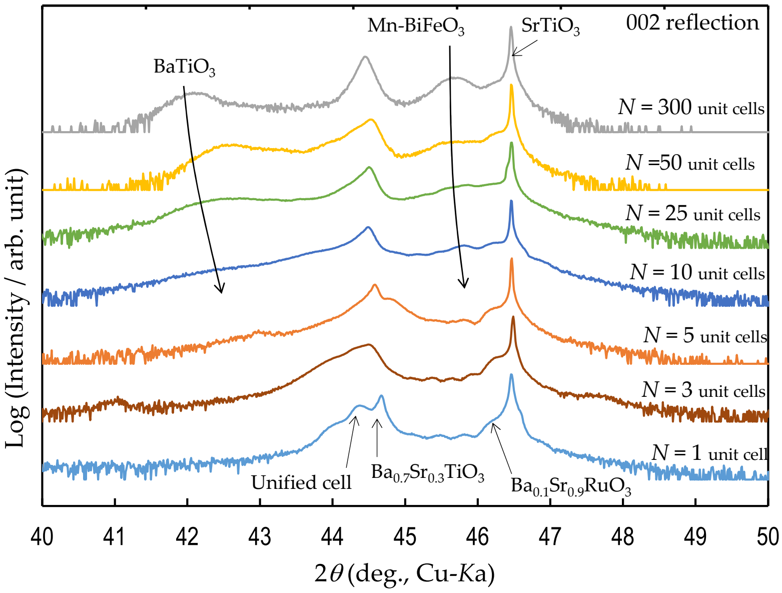

Figure S1. θ-2θ XRD patters around 002 reflection; N denotes the number of ABO_3 unit cells in the two layers of Mn-$BiFeO_3$ and $BaTiO_3$ comprising the superlattice. The unified cell indicates a structurally and electronically coupled unit cell composed of the Mn-$BiFeO_3$ and $BaTiO_3$ layers.

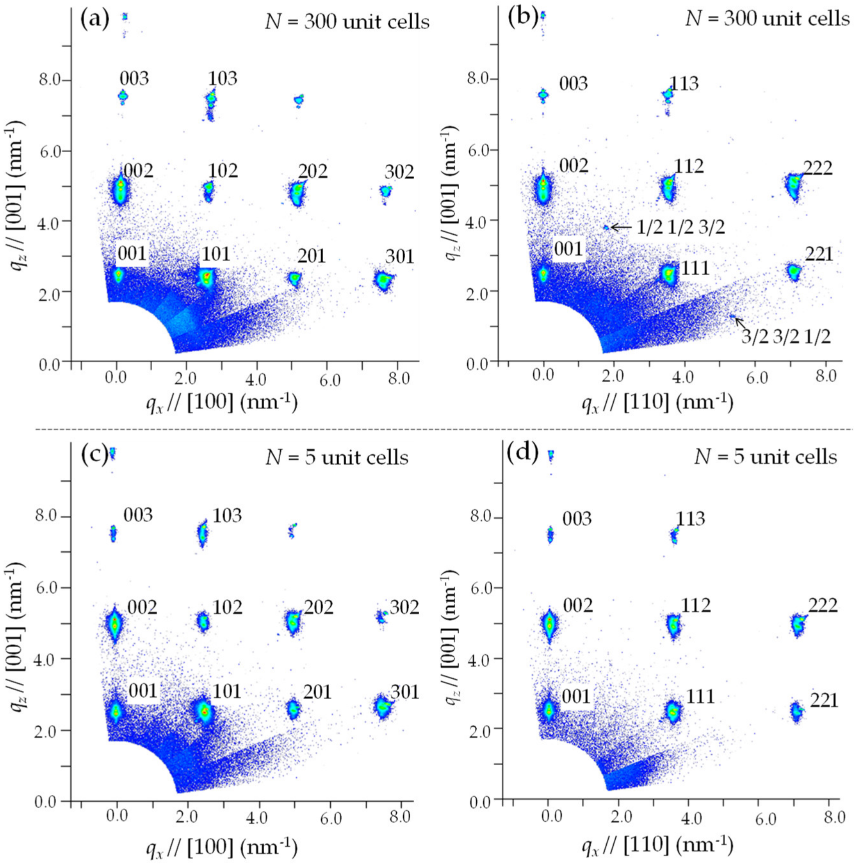

Figure S2. Wide-area XRD-RSMs for (**a**,**b**) $N = 300$ and (**c**,**d**) $N = 5$. The vertical axis is q_z // [001] throughout. The horizontal axis is q_x // [100] for (**a**) and (**c**) and q_x // [110] for (**b**) and (**d**). The [001] and [100] are the crystallographic directions of the (100) $SrTiO_3$ substrate.

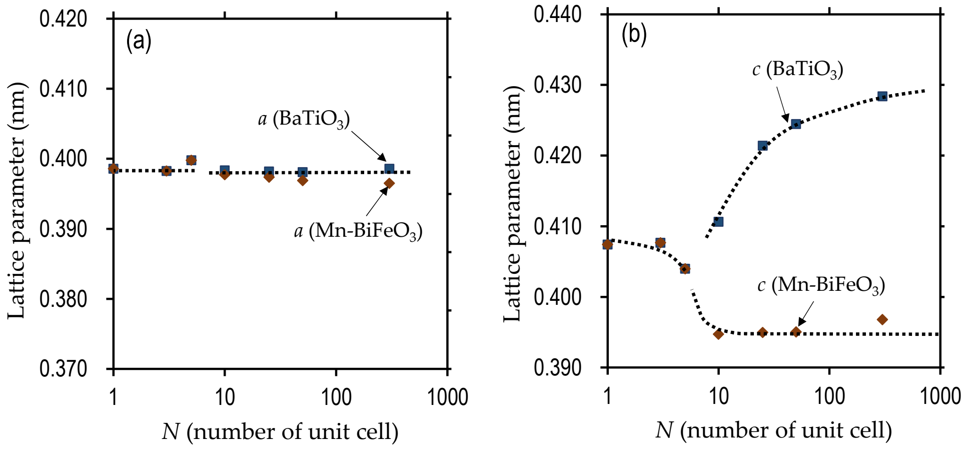

Figure S3. Lattice parameters estimated from the peak positions of 103 reflection in the high-resolution XRD-RSMs as a function of *N*, where *N* denotes the number of ABO_3 unit cells in the two layers of Mn-$BiFeO_3$ and $BaTiO_3$ comprising the superlattice. As described in Supplementary Note 3, we have to address an inevitable alignment issue in the XRD measurements: the reflection from the $BaTiO_3$ layer cannot be observed precisely and thereby the hem of its 103 reflection is detected, as displayed in Figs. 4(a) and (b). It causes the overestimation of the parameter $c(BaTiO_3)$.

Supplementary Note S1. Deposition Conditions of PLD

The deposition conditions for the $Ba_{0.7}Sr_{0.3}TiO_3$ buffer layer, the $Ba_{0.1}Sr_{0.9}RuO_3$ electrode, the Mn (5 %)-$BiFeO_3$ layer, and the $BaTiO_3$ layer are summarized in Table S1. We confirmed that the number of laser shots to obtain one ABO_3 unit cell (u.c.) of Mn (5 %)-$BiFeO_3$ and $BaTiO_3$ are 16 shots/u.c. and 7 shots/u.c., respectively. Based on these results, we control the number of ABO_3 unit cells in each layer comprising the superlattices, e.g., for the sample with $N = 3$ (three ABO_3 unit cells in each layer), the Mn-$BiFeO_3$ layer is fabricated by 16 × 3 laser shots and the $BaTiO_3$ layer by 7 × 3 laser shots, as listed in Table S2.

Table S1. Deposition conditions of substrate temperature (T_{sub}), oxygen partial pressure (P_{O2}), laser repetition frequency, and laser fluence. Information on the thickness of the $Ba_{0.7}Sr_{0.3}TiO_3$ buffer layer and the $Ba_{0.1}Sr_{0.9}RuO_3$ electrode is listed. .

Target	T_{sub} (°C)	P_{O2} (Pa)	Repetition Frequency (Hz)	Laser Fluence (J/cm²)	Thickness (nm)
$Ba_{0.7}Sr_{0.3}TiO_3$ *	740	0.26	1	1.2	200
$Ba_{0.1}Sr_{0.9}RuO_3$	610	13	1	1.2	30
Mn (5 %)-$BiFeO_3$	640	2.6	7	1.5	—
$BaTiO_3$	640	2.6	1	1.5	—

* We used the target with the 5%-Ti-excess composition of $Ba_{0.7}Sr_{0.3}Ti_{1.05}O_3$ (Noguchi, Y., Maki, H., Kitanaka, Y., Matsuo, H. & Miyayama, M. Appl. Phys. Lett. 113, 012903 (2018)).

Table S2. Number of laser shots to deposit one layers of Mn-$BiFeO_3$ and $BaTiO_3$ comprising the superlattice samples.

N (Unit Cell)	Number of Laser Shots for One Layer (–)	
	Mn (5 %)-$BiFeO_3$	$BaTiO_3$
300	4,800	2,100
50	800	350
25	400	175
10	160	70
5	80	35
3	48	21
1	16	7

Supplementary Note S2. Crystal Structural Analyses for the Mn (5%)-$BiFeO_3$ Layer

Supplementary Figure S4a shows the X-ray diffraction (XRD) reciprocal space map (RSM) around 113 reflection for $N = 300$. It seems that the reflection from the Mn-$BiFeO_3$ layer splits into two peaks that are positioned at different q_x with almost the same q_z. This result can be qualitatively explained by a presence of ferroelastic domains (Supplementary Figure 4b) in rhombohedral $R3c$ symmetry. Moreover, we carefully investigate the possibility of the monoclinic M_A or M_B structure (the lattice parameters: a_m, b_m and c_m, and the reciprocal vectors: a_m^*, b_m^* and c_m^*) that is distorted from the rhombohedral $R3c$. Considering Domain 1 with the reciprocal vectors ($a_{m,1}^*$, $b_{m,1}^*$and $c_{m,1}^*$) and Domain 2 with those of $a_{m,2}^*$, $b_{m,2}^*$, and $c_{m,2}^*$, as shown Supple. Fig. 4(d) and (e), we obtain $a_m = 0.5631$ nm, $b_m = 0.5573$ nm, $c_m = 0.7933$ nm, and $\gamma = 89.7$ °. From these results, we think that the Mn-$BiFeO_3$ layer is in a monoclinic phase that is slightly distorted from the rhombohedral $R3c$.

The pseudocubic lattice parameters a and c expressed as $a = \sqrt{(a_m{}^2 + b_m{}^2)/2}$ and $c = c_m/2$ are 0.3961 nm and 0.3966 nm, which are quantitatively in good agreement with the results show in Supple. Fig. 3. Because of $a < c$ we conclude that the Mn-$BiFeO_3$ layer for

N = 300 has the monoclinic M_A structure with quite small distortion from the rhombohedral $R3c$.

On one hand, the relation between the parameter a and c is reversed in the range of 50 ≥ N ≥ 10, i.e., $a > c$ (Supplemantary Figure S3) owing to a tensile strain from the $BaTiO_3$. This result indicates that the Mn-$BiFeO_3$ layer has the monoclinic M_B phase whose distortion from the rhombohedral structure is quite small. The absence of the peak splitting of the 103 reflection from the Mn-$BiFeO_3$ layer for N = 25 and 10 [Figure 4c,d) displays that a deviation from the tetragonal structure is small in the Mn- $BiFeO_3$ layer, as depicted in Supplementary Figure S5b.

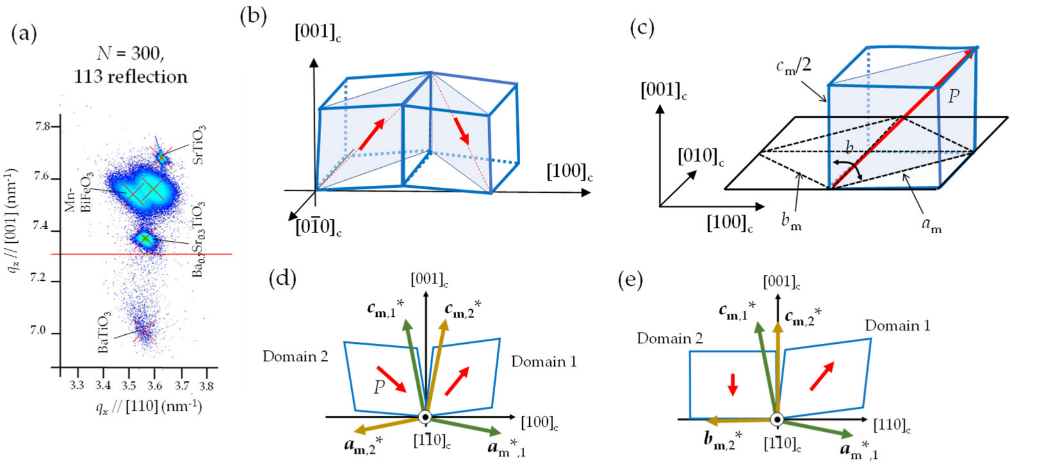

Figure S4. (**a**) High-resolution XRD-RSM around 113 reflection for N = 300 where the vertical axis is q_z // [001] and the horizontal axis is q_x // [110]. Schematics of (**b**) ferroelastic domain structure, (**c**) the crystal structure of the M_A phase, and reciprocal lattice vectors of two domains comprising the ferroelastic domain structure projected onto (**d**) $[100]_c$ vs $[001]_c$ plane and (**e**) $[110]_c$ vs $[001]_c$ plane.

Supplementary Note S3. Crystallographic Relation between the Monoclinic Mn-$BiFeO_3$ Layer and the Tetragonal $BaTiO_3$ Layer

During the deposition of superlattices at a substrate temperature (T_{sub}) of 640 °C which is lower than the Curie temperature of around 800 °C for Mn-$BiFeO_3$ (S. Basu et al., *Curr. Appl. Phys.* **11,** 976–980 (2011)), the $BaTiO_3$ layer is deposited on the Mn-$BiFeO_3$ layer with a rhombohedral-like monoclinic distortion via a heteroepitaxial growth, as shown in Supple. Fig. 5(a). For N = 300 and 50, the 103 reflection of the Mn-$BiFeO_3$ layer has two peaks because of the rhombohedral-like monoclinic distortion with the ferroelastic 109 ° domains. This Mn-$BiFeO_3$ structure causes a deviation of the tetragonal axes of the $BaTiO_3$ (BTO) layer, the $[100]_{BTO}$ and the $[001]_{BTO}$, from the $[100]_c$ of the $SrTiO_3$ substrate. In the measurement system where the alignment is strictly performed based on the standard axes of the substrate, the reflection from the $BaTiO_3$ layer cannot be observed precisely and thereby the hem of its 103 reflection is detected, as displayed in Figure 6a,b. This inevitable alignment issue is the reason of the overestimation of the parameter c($BaTiO_3$) (Supplementary Figure S3).

In the range of 50 ≥ N ≥ 10, the Mn- $BiFeO_3$ layer has a small deviation from the tetragonal structure due to a less structural distortion, and therefore the $[100]_{BTO}$ and the $[001]_{BTO}$ become close to the standard $[100]_c$ and $[001]_c$ of the substrate [Supplementary Figure S5b, respectively.

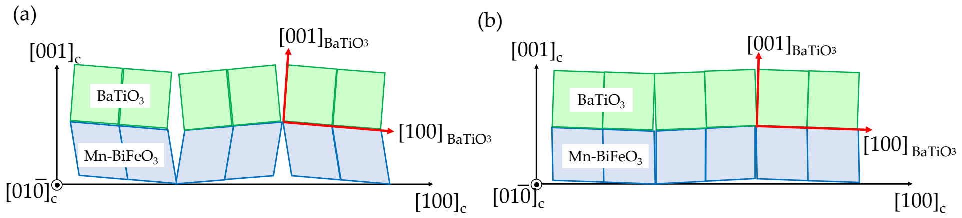

Figure S5. Relationships between the rhombohedral-like monoclinic Mn-$BiFeO_3$ layer and the tetragonal $BaTiO_3$ layer (a) for $N = 300$ and (b) for $50 \geq N \geq 10$ where the rhombohedral feature of the Mn-BFO layer becomes weak. The slanted [001] and [100] axes of the $BaTiO_3$ layer are indicated as $[001]_{BTO}$ and $[100]_{BTO}$, respectively.

Supplementary Note S4. Superlattice Structures and Their Ambiguity

At present, we cannot provide direct, real-space evidence, e.g., transmission electron microscope image, of the superlattice structures in our samples. Nevertheless, we think that the *in-plane average* structure can be regarded as its designed one delivering the unique polarization and dielectric properties (Figures 5 and 6) owing to the following reasons. All the superlattice samples exhibit a ferroelectric polarization hysteresis with an apparent remanent polarization (P_r) over 15 μC cm^{-2} for N of 10 or less If the structure of the sample were not the superlattice and were similar to that in a solid solution of 50% Mn-$BiFeO_3$ and 50% $BaTiO_3$, the P_r should be zero because of its non-ferroelectric nature (Leontsev, S. O. & Eitel, R. E. *J. Am. Ceram. Soc.* **92**, 2957–2961 (2009)). Moreover, the DFT-derived spontaneous polarization (P_s) and its dependence on N (Figure 6d) are quantitatively in good agreement with the experiments. Our property experiments and theoretical calculations support that the designed structures depicted in Figure 1 are maintained in an *in-plane average* structure of our superlattice samples. As for the samples with N greater than 25, the simultaneous presence of the reflections of 3/2 3/2 1/2 and 1/2 1/2 3/2 originating from the rhombohedral-like monoclinic Mn-$BiFeO_3$ layer (Figure 3) and that of the 103 reflection from the $BaTiO_3$ layer with a relatively large tetragonal distortion (c/a) is direct, reciprocal-space evidence that the samples have a superlattice composed of Mn-$BiFeO_3$ and $BaTiO_3$.
